# Editorial: New clinically important *Candida* species and other emerging yeasts: navigating new challenges in antifungal resistance, diagnosis and treatment

**DOI:** 10.3389/fcimb.2026.1910193

**Published:** 2026-07-01

**Authors:** María Guadalupe Frías-De-León, Eduardo García-Salazar

**Affiliations:** Laboratorio de Micología Molecular, Unidad de Investigación Biomédica, Hospital Regional de Alta Especialidad Ixtapaluca, Servicios de Salud del Instituto Mexicano de Seguro Social para el Bienestar (IMSS-BIENESTAR), Ixtapaluca, Mexico

**Keywords:** *C. auris*, Candidemia, diagnostics, training of healthcare workers, treatment

The incidence of fungal diseases has increased in the last two decades driven by a expanding pool of vulnerable populations and the compounding effects of climate change. Among these mycoses, candidiasis remains a critical concern due to its high mortality rates in invasive forms. In late 2022, the World Health Organization published a list of priority fungal pathogens based on their incidence and resistance to antifungals. Within this list, *Candida auris* (*Candidozyma auris*) and *C. albicans* were designated as critical priority; *C. glabrata* (*Nakaseomyces glabrata*), *C. tropicalis*, and *C. parapsilosis* as high priority; and *C. krusei* (*Pichia kudriavzevii*) as medium priority. Among the main objectives of this classification are to promote the development of novel antifungals and to improve the healthcare system’s capacity to detect and identify these pathogens and contain outbreaks early. Based on this, in this Research Topic we aimed to present some of the latest advances in the diagnosis and treatment of *Candida* infections.

The timely diagnosis of invasive candidiasis, particularly candidemia, remains one of the greatest challenges in medical mycology. The limited sensitivity of blood cultures—the current diagnostic gold standard—and the constraints of culture-dependent identification methods often delay appropriate treatment, compromising patient outcomes. Against this backdrop, the development of rapid, culture-independent diagnostic tools represents a significant advance.

A significant contribution to this research area is the study by Wang et al., which presents an innovative approach for the direct detection and identification of *Candida* species in blood samples. Their method combines recombinant human mannan-binding lectin microspheres (M1 microspheres) for *Candida* enrichment in blood with a recombinase-assisted multiplex PCR (mRAP) assay for sensitive DNA amplification. This strategy allows for the detection of *C. albicans*, *C. tropicalis*, and *C. glabrata* at low concentrations—down to 2, 2, and 1 CFU/mL, respectively—in less than four hours. Since rapid diagnosis is essential for the immediate initiation of antifungal therapy, the M1-mRAP method may be useful for improving clinical decision-making.

Among the etiological agents of candidemia, *C. auris* has emerged as a major global health concern due to its multidrug-resistant, which complicates patient management, and contributes to elevated high mortality rates. The need for effective antifungal alternatives has driven research into new compounds with antifungal potential. In this context, Yang et al. propose the use of biocontrol agents, such as *Bacillus velezensis* NC-B4, which possesses cellulase and protease activity, as well as some antibacterial secondary metabolites that inhibit the growth of *C. auris* and *C. albicans*, and the formation of biofilms. These findings reveal that *B. velezensis* NC-B4 can serve as a basis for the development of new antifungals.

Another innovative approach presented in this Research Topic involves the application of titanium dioxide (TiO_2_) nanoparticles. Gómez-Hernández et al. demonstrated that TiO_2_ nanoparticles, at concentrations of 1, 10, and 50 µg/mL, exert an antifungal effect against C. glabrata, both in its planktonic form and internalized in macrophages. These findings suggest that TiO_2_ nanoparticles may represent another alternative against fungal infections caused by resistant *Candida* species.

Among the alternative therapeutic approaches highlighted in this Research Topic, traditional Chinese medicine (TCM) stands out as a promising source of antifungal molecules. Zhu et al. evaluated the antimicrobial activity of the TCM multiherbal solution MangHuang (MH) against *C. albicans*. The findings of the study showed significant inhibitory effect on biofilm formation, mediated by a multi-target mechanism that affect intracellular metabolic processes. This multi-target mechanism is relevant as it may reduce the likelihood of resistance development by simultaneously targeting multiple cellular pathways. Therefore, further studies are needed to characterize the specific bioactive compounds contained in the MH formulation and to validate the results in physiological models.

On the other hand, attention must also be directed toward emerging fungal pathogens with concerning resistance profiles, as *Diutina catenulata* (formerly *Candida catenulata*), a yeast that exhibits a remarkably high antifungal resistance profile. Zhang et al. evaluated the antifungal susceptibility of *D. catenulata* and found high resistance to fluconazole and caspofungin in strains isolated from China. The *D. catenulata* isolates showed greater resistance than that commonly observed in other species, such as *C. albicans*. These findings suggest possible adaptive evolution under the pressure of natural selection and underscore the urgent need for regional resistance surveillance.

Finally, an important aspect of managing infections caused by emerging and multidrug-resistant yeasts is preparing healthcare personnel to recognize and respond to potential outbreaks. In this context, Khánh Huy et al. assessed the knowledge, attitudes, and practices related to *C. auris* among healthcare professionals working in the intensive care unit (ICU) of a private hospital in Hanoi, Vietnam. Despite the high mortality rate associated with *C. auris* in ICUs, the findings of this study showed that healthcare professionals’ knowledge about *C. auris* is limited. Given that similar deficiencies may exist in other low- and middle-income countries, it is recommended that relevant training programs be designed for healthcare professionals in ICUs, in order to better prepare both the public and private health systems to support timely responses to outbreaks caused by this multidrug-resistant yeast.

In conclusion, new therapeutic approaches range from biological control agents to nanomaterials and traditional herbal formulations, expanding treatment options against resistant *Candida* species. Diagnostic tools, such as the M1-mRAP assay, offer rapid and accurate detection of candidemia caused by some of the most clinically relevant *Candida* species. However, it will be necessary to broaden the range of identified species to include emerging multidrug-resistant pathogens, such as *D. catenulata*. Taken together, these advances underscore the importance of integrating improved diagnostics, innovative therapies, resistance surveillance, and healthcare worker training to address the growing challenge of invasive *Candida* infections worldwide.

